# A Pilot Study of Children’s Blood Lead Levels in Mount Isa, Queensland

**DOI:** 10.3390/ijerph14121567

**Published:** 2017-12-13

**Authors:** Donna Green, Marianne Sullivan, Nathan Cooper, Annika Dean, Cielo Marquez

**Affiliations:** 1 Climate Change Research Centre, University of New South Wales, Kensington, Sydney, NSW 2052, Australia; n.cooper@unsw.edu.au (N.C.); annika.dean@unsw.edu.au (A.D.); 2 The ARC Centre of Excellence for Climate Systems Science, University of New South Wales, Kensington, Sydney, NSW 2052, Australia; 3 Department of Public Health, William Paterson University, Wayne, NJ 07470, USA; sullivanm19@wpunj.edu; 4 Sonic HealthPlus Super Clinic, Ryan Road, Mount Isa, QLD 4825, Australia; sky_cielo01@yahoo.com

**Keywords:** children’s blood lead level, Mount Isa, lead smelter, Indigenous, LeadCare II Point of Care

## Abstract

Mount Isa, Queensland, is one of three Australian cities with significant lead emissions due to nonferrous mining and smelting. Unlike the two other cities with lead mines or smelters, Mount Isa currently has no system of annual, systematic, community-wide blood lead level testing; and testing rates among Indigenous children are low. In previous screenings, this group of children has been shown to have higher average blood lead levels than non-Indigenous children. The first aim of this study was to assess whether parents and children would participate in less invasive, rapid point-of-care capillary testing. The second aim was to measure blood lead levels among a range of children that roughly reflected the percentage of the Indigenous/non-Indigenous population. This pilot study is based on a convenience sample of children between the ages of 12 and 83 months who were recruited to participate by staff at a Children and Family Centre. Over three half-days, 30 children were tested using capillary blood samples and the LeadCare II Point-of-Care testing system. Rapid point-of-care capillary testing was well tolerated by the children. Of 30 children tested, 40% (*n* = 12) had blood lead levels ≥5 µg/dL and 10% had levels ≥10 µg/dL. The highest blood lead level measured was 17.3 µg/dL. The percentage of children with blood lead levels ≥5 µg/dL was higher among Indigenous children compared to non-Indigenous (64.2% compared to 18.8%) as was the geometric mean level (6.5 (95% CI, 4.7, 9.2) versus 2.4 (95% CI, 1.8, 3.1)), a statistically significant difference. Though based on a small convenience sample, this study identified 12 children (40%) of the sample with blood lead levels ≥5 µg/dL. Due to historical and ongoing heavy metal emissions from mining and smelting in Mount Isa, we recommend a multi-component program of universal blood lead level testing, culturally appropriate follow-up and intervention for children who are identified with blood lead levels ≥5 µg/dL. We further recommend focused outreach and assistance to the Indigenous community, and further control of emissions and remediation of existing environmental lead contamination in children’s play and residential areas.

## 1. Introduction

**Figure 1:**
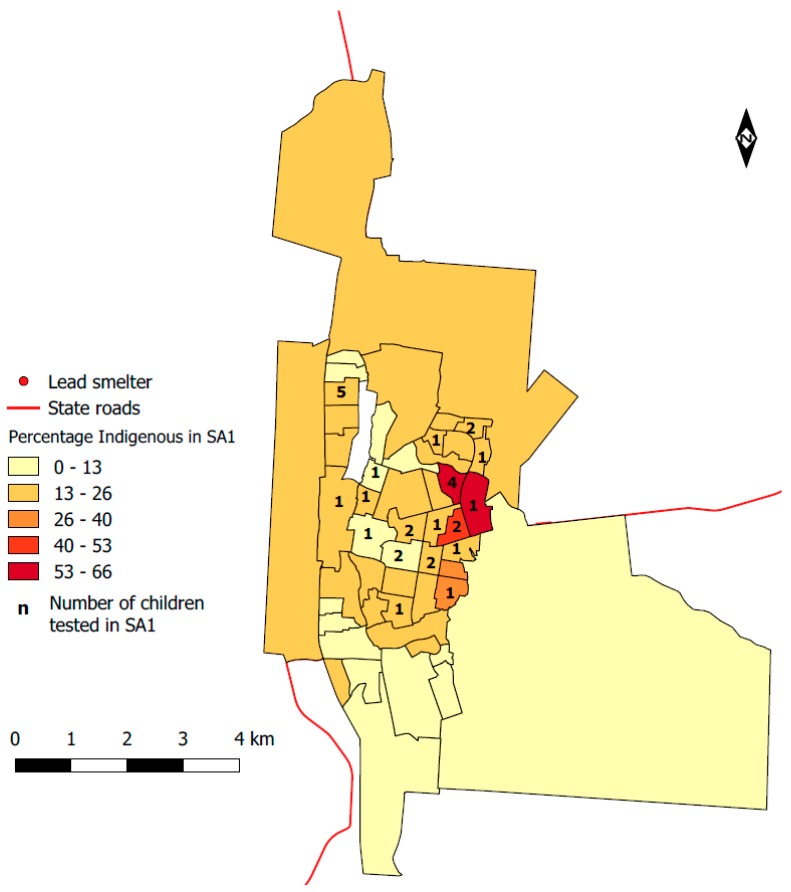
Map of Mount Isa with percentage of residents of Indigenous status and the number of children tested for blood lead levels in this study by Statistical Area 1 (SA1).

The city of Mount Isa is the most important industrial, commercial, and administrative centre in outback Queensland. Founded nearly a century ago on land belonging to the Kalkadoon Aboriginal tribe, the large inland city’s economy depends on the employment and industry that exists around the lead and copper smelter, as well as several mines from which the ore that feeds the smelters is extracted. The city developed in proximity to the smelters and mines (see Figure 1). According to the National Pollution Inventory, the Mount Isa Mines emitted 81,000 kg of lead in 2013/2014 [1] and 18,000 kg in 2015/2016 as air releases [2]. The mining and smelting complex is also a significant source for other toxic metals [3].

Starting with a handful of the early prospectors who found ore in 1923, Mount Isa has grown to a city of over 22,000 people, just over 8% of whom are children between the ages of 0 and 4, and 16% of whom are children aged 0–9 [4]. Mount Isa is an important service centre for several Indigenous communities spread across a very large region of the state. The city is situated on, and surrounded by, Indigenous land. As a result, there is a higher than average Indigenous population in the city compared to the rest of the state. Almost 20% of the city is Indigenous, compared to the state average of 5% [5,6]. The ‘gap’ for Indigenous people in terms of social, economic and health and well-being observed in Australia is clearly seen in Mount Isa [7]. For example, the difference between Indigenous and non-Indigenous median age at death is 13 years (53 and 66 years, respectively) [6].

Childhood lead exposure in nonferrous mining and smelting communities worldwide has been an ongoing concern since its recognition by public health researchers in the early 1970s [8]. Decades of research have shown that children living near nonferrous mines and smelters are at risk of lead exposure and that proactive environmental and public health strategies are needed to monitor and reduce exposure [8,9,10,11,12,13,14]. Research in Mount Isa has documented elevated concentrations of lead in soil, dust and air as a result of mining and nonferrous metal smelting [15].

Both Australia and the US have lowered the blood lead level in children at which public health intervention should begin, from 10 to 5 µg/dL due to the accumulating body of scientific evidence on the effects of low-level lead [16,17]. A 2012 review of the scientific literature published by the US National Toxicology Program concluded that there is “sufficient evidence that blood Pb levels <10 and <5 µg/dL are associated with adverse health effects in children and adults” [18]. Health effects associated with childhood lead exposure at blood lead levels <5 µg/dL include “increased diagnosis of attention-related behavioral problems, greater incidence of problem behaviors, and decreased cognitive performance” [18]. At blood lead levels <10 µg/dL, lead exposure is associated with “delayed puberty and reduced postnatal growth” [18]. Among pregnant women, blood lead levels <5 µg/dL are associated with reduced fetal growth, and blood lead levels <10 µg/dL are associated with increased “spontaneous abortion and preterm birth” [18]. A recent US EPA Integrated Science Assessment determined that there is a causal relationship in children between lead exposure and IQ decrements and behavioral problems such as “attention and impulsivity and hyperactivity” [19].

There is a strong scientific consensus that exposure to low-level lead has serious adverse effects on children’s health and development and should be prevented [17]. Nonferrous mines and smelters are well-recognised sources of exposure [20]. Preventing and ameliorating childhood lead exposure near these sources requires a strong regulatory framework and enforcement, monitoring and remediation of pollution, and universal blood lead testing of children to identify children in need of public health and/or medical intervention.

Concerns about childhood lead exposure in Mount Isa are long-standing. For example, a Queensland Health report noted that a government testing effort in 2006–2007 was conducted in response ‘to concerns about community exposure to lead’ [21]. Other concerns have centered on low rates of testing [22,23], high blood lead levels in some children [24], perceived ‘complacency’ of governmental health leadership [25], disproportionate impacts on Indigenous children [26], and the accuracy of health education information provided to the community and its cultural appropriateness [27].

Despite these concerns, a large-scale, annual, and systematic screening program has not been implemented in Mount Isa unlike in Port Pirie and Broken Hill. For example, in Broken Hill, blood lead levels are tested annually in a significant proportion of the 1–4 population, and the results are published in a detailed annual report. In 2015, 77% (*n* = 679) of all children ages 1–4 were screened in Broken Hill, and the screening rates for Indigenous children exceeded 100% (potentially due to under-identification of Indigenous status in the Census which serves as the denominator, or in-migration of Indigenous child residents) [28].

South Australia Health publishes detailed reports on a quarterly and annual basis on children’s blood lead levels in Port Pirie. In 2016, 641 children under the age of 5 were tested, including 113 children 24 months of age [29]. Because of limitations of census data, it is not possible to determine precisely, but approximately two-thirds of the 0–4 population of Port Pirie is tested annually.

In Mount Isa, the proportion of children tested annually is much lower. For example, based on testing and denominator data provided by Queensland Health, in 2012, only 2.2% of the under 5 population was tested. The highest rate of testing was in 2014 (8%), and in the most recent data available (2015), 7.7% of Mount Isa children ages 1–4 were tested [22]. More recently, based on a media report, it appears that approximately 25% of Mount Isa children were tested in the past year; however, these data have not been publicly released [30].

The limited blood lead level data for Mount Isa children make it difficult to assess the dimensions of the problem. Despite this, the data show a persistence of blood lead levels ≥5 µg/dL in the community since the early 1990s when testing began. The earliest screening, conducted by the Mount Isa Mines between 1992 and 1994, was based on a non-random sample of 101 children. More than one-third (36.7%) of tested children had blood lead levels greater than 10 µg/dL, and 15.6% of children tested had blood lead levels greater than 15 µg/dL [31] as can be seen in Table 1.

The largest testing effort in Mount Isa occurred in 2006–2007 and was conducted by Queensland Health. This screening included 400 children ages 1–4 who ‘were not randomly selected but were invited to participate’ [31] but whose age, gender, and Indigenous status matched that of the overall population of children in that age group in Mount Isa. Venepuncture was the method used for blood collection in this study. Eighty-three of those tested were Indigenous children, 11.3% of whom had blood lead levels ≥10 µg/dL and Indigenous children had a statistically significantly higher geometric mean blood lead level (7 µg/dL compared to non-Indigenous children (4.5 µg/dL)) [31].

A smaller Queensland Health screening, again based on self-selection, in 2010, included 167 children, 37 of whom were identified as Indigenous. Blood samples were collected through venepuncture. In this sample, 4.8% of children had blood lead levels ≥10 µg/dL and Indigenous children were again found to have statistically significantly higher geometric mean blood lead levels compared to non-Indigenous children (5.4 µg/dL compared to 4 µg/dL) [21].

Aside from these two detailed reports on children’s blood lead levels in Mount Isa [21,31], more recent publicly available data are based on a limited number of children tested, and lack important details such as the number of Indigenous participants, the distribution of blood lead levels in Indigenous and non-Indigenous children, the highest blood lead level measured, and blood lead levels by age and gender. No testing data were reported for 2011, but in each year of data since 2012, the number of Indigenous children tested has not been reported, making it impossible to assess rates of screening in this population. Geometric mean blood lead levels in these opportunistic samples have ranged from 2.6 to 3.2 µg/dL in the overall population of children tested and from 3.0 to 3.9 µg/dL among Indigenous children [22].

In the first seven months of 2017, two newspaper articles reported on blood lead level screening results in Mount Isa. The first from February 2017 stated that according to Queensland Health, in 2016 the mean blood lead level was 2.4 µg/dL overall and 2.9 µg/dL among Indigenous children. Five children (3% of those tested) were said to have blood lead levels >10 µg/dL and 25 (6.6%) were reported to have blood lead levels >5 µg/dL [32]. From these data, we infer that the total number of children tested in 2016 was 166. In July 2017, a North West Star article reported that Dr Jeanette Young, Queensland’s Chief Health Officer, provided data for September 2016–June 2017. Five hundred and seventy children were tested during this time period, approximately 25% of the 1–4 year old population of Mount Isa. The mean blood lead level was reported as 2.3 µg/dL and no data were provided for Indigenous children. One hundred thirty-one children (23%) were reported to have blood lead levels >5 µg/dL, and no data were provided on children with blood lead levels ≥10 µg/dL. We have been unable to find an official Queensland Health report on these recent data. Despite the fact that nearly one-quarter of children tested were reported to have blood lead levels >5 µg/dL, the article headline read “Good Results in Latest Round of Mount Isa Testing” [30].

There are four important problems with current blood lead level screening in Mount Isa: due to the small proportion of the population of 1–4-year-olds tested and the lack of random sampling, the approach does not allow for valid inferences to be drawn about whether or not children’s blood lead levels are improving, staying the same, or worsening; Indigenous children appear to be under-represented and there is evidence they are at highest risk of blood lead levels ≥5 µg/dL; and, not all children with blood lead levels ≥5 µg/dL are being identified, which means that critical intervention to reduce blood lead levels and prevent further exposure is not occurring. Finally, data are not fully and publicly reported in detailed technical reports as they are in Port Pirie and Broken Hill. Despite these problems with data, Queensland Health says blood lead levels in Mount Isa children are ‘falling’ [32,33].

A range of governmental and non-governmental representatives have called for increased blood lead level testing in Mount Isa. In an annual report, the Living with Lead Alliance “recommends that all Mount Isa residents children and adults know their BLLs” [34], and the Commissioner for Children and Young People has also called for increased testing [22]. The recently published ‘Lead Pathways Study-Air’ [35] also stressed the importance of increased screening, as have other public health and environmental experts [36]. Despite the reported increase in testing in the past year, an important improvement over other years, testing rates in Mount Isa remain low.

The first aim of this study was to assess whether parents and children would participate in less invasive, rapid capillary testing at a community location (a Children and Family Centre). The second aim was to measure blood lead levels among a range of children that roughly reflected the percentage of the Indigenous/non-Indigenous population.

## 2. Materials and Methods

### Study Design

This pilot study tested blood lead levels in a convenience sample of children ages 1–6 years who had lived in Mount Isa for at least three months prior to the time of testing. The study was carried out at a community Children and Family Centre in Mount Isa which is located in an area of the city with a large Indigenous population. We used capillary sampling and the LeadCare II Point-of-Care testing system to provide rapid blood lead level results. LeadCare II is a US Food and Drug Administration (FDA)-approved blood lead testing device waived by the Clinical Laboratory Improvement Amendments (CLIA) of 1988. The CLIA waiver indicates that, due to its low complexity and accuracy, it can be used at non-traditional testing sites [37,38]. LeadCare II uses anodic stripping voltammetry to measure lead in blood samples. The limits of detection for LeadCare II are 3.3–65 µg/dL. The reliability and validity of LeadCare II has been established in other studies, and it is used worldwide for blood lead screening [37,38]. Prior to testing children, all study personnel completed online training and earned the LeadCare training completion certificate.

Recruitment was conducted by Centre staff using word of mouth. Prospective parent participants were told that blood lead testing would involve a ‘finger prick’ test, and results would be available immediately. A $50 gift card would be given at the end of the test as compensation for their time. Blood lead testing occurred on three mornings, one in March 2017 and two on days in May 2017.

Prior to testing, the study was explained to parents, and all parents provided written informed consent. All tests took place in a clinical examination room. We took steps to minimise the possibility of external lead contamination in the examination room. Surface areas on which blood lead testing supplies were kept during testing sessions were cleaned, and a clean barrier cloth was laid down prior to testing. Alcohol prep pads and gauze were unopened until use, and capillary tubes and sensor strips were kept in the manufacturer’s containers with lids closed until use.

To minimise the possibility of external contamination from the children’s skin during capillary sampling, all children’s hands were thoroughly washed with soap and water immediately prior to puncturing the skin. After handwashing, a clean, disposable white paper towel was used to dry the children’s hands. Then, the child’s finger was wiped with alcohol and dried with sterile gauze. Non-reusable 1.8 mm lancets were used to puncture the skin, and the first drop of blood was wiped away. Capillary blood was drawn by the same local doctor, wearing lead-free examination gloves during all three testing sessions. Blood samples were collected in LeadCare provided capillary tubes and were mixed by inverting the tubes multiple times in LeadCare reagent tubes. Once the blood–reagent mixture turned brown, it was tested in the LeadCare machine. Only one lot of testing supplies was used in this study. Two sets of controls were run three times to verify that the machine was working properly and after the machine was moved prior to testing (twice). Manufacturer’s instructions were followed for running controls; all three sets of controls were within the manufacturer’s provided acceptable limits.

While the child’s blood sample was being tested, parents/guardians were asked a short set of questions about: their child’s age, gender, Indigenous status, and history of previous blood lead level testing, their type of residence, the time their children spent playing outdoors, and their garden characteristics (grass, bare earth, etc.).

The result of the child’s lead test was immediately reported to the parent by the doctor, recorded for research purposes, and provided to the parent in writing along with lead health education resources. The doctor answered any questions parents had about their child’s blood lead level at the time the blood lead result was provided. Children with blood lead levels ≥5 µg/dL were advised to take their child for confirmatory venous testing within a month. The doctor also kept a record of blood lead levels ≥5 µg/dL and followed up with parents/guardians to recommend confirmatory testing or other medical follow-up.

To calculate the percentage of residents of Indigenous status within each SA1, we downloaded census data for Mount Isa from the most recent census. The total number of Indigenous residents in each SA1 was determined by calculating the sum of people who were listed as ‘Aboriginal’, ‘Torres Strait Islander’, or both in the census’ Indigenous status question. We calculated the total population by summing the calculated Indigenous population with the population listed as ‘Non-Indigenous’ on the Indigenous status question, and used this figure to calculate the proportion of the total population in each SA1 that consisted of Indigenous residents.

Of the 50 geographic units in Mount Isa, only one did not have census counts for either Indigenous or non-Indigenous residents and therefore, a percentage could not be calculated. This SA1 was not categorised in the colour scheme used for all other SA1s in Figure 1 to designate the percentage of residents of Indigenous status. Each SA1 also contained a number of people who did not answer the Indigenous status census question. Since we could not determine their status, we did not include them in our calculation.

Data were entered into Excel and then imported into SPSS and R for analysis. The analysis consisted of descriptive statistics, calculating geometric mean blood lead level, and testing population variables for statistical significance with blood lead levels. Levels below LeadCare’s limits of detection were reported as <3.3 µg/dL. For the summary statistics, blood lead levels recorded as <3.3 µg/dL were replaced with the value 1.6 (limit of detection/2), appropriate in this case, as our data are highly skewed [39]. The geometric mean was calculated for the entire dataset of blood lead levels, as well as for each categorical variable. The geometric mean is a better representation of the average value of a dataset compared to the arithmetic mean when the dataset is highly skewed and therefore does not follow a normal distribution.

This research was approved by the HREA Panel E: HC16398, University of New South Wales, Australia and the Institutional Review Board of William Paterson University of New Jersey, USA.

## 3. Results

In three half-days of testing, we measured blood lead levels in 30 children. Sample characteristics are described in Table 2. The majority of the sample (93%) was between 1 and 4 years of age; the youngest child was 12 months and the oldest was 6 years. Sixty percent of children who participated were female, and 47% were self-identified as Indigenous.

Thirty-six percent of those tested (11 children) had blood lead levels below the limits of detection (<3.3 µg/dL), and 60% (18 children) had blood lead levels <5 µg/dL. However, 12 children, or 40% of the sample, had blood lead levels ≥5 µg/dL. Ten percent of those tested had blood lead levels ≥10 µg/dL. The highest blood lead level measured in this study was 17.3 µg/dL. Of the 14 Indigenous children who participated, 9 (64.2%) had blood lead levels ≥5 µg/dL. Among non-Indigenous children the percentage was lower, 18.8% had blood lead levels ≥5 µg/dL (see Table 3).

The geometric mean blood lead level in the overall sample was 3.8 µg/dL (95% CI, 2.9, 5.0) and was statistically significantly higher for Indigenous children (6.5 µg/dL (95% CI, 4.7, 9.2)) than for non-Indigenous children (2.4 µg/dL (95% CI, 1.8, 3.1)). The majority of children who participated in this study (93%) had not previously been tested for lead exposure.

With respect to the acceptability of capillary testing, the method was well received by parents who appeared to appreciate receiving immediate results and having the opportunity to discuss them with the doctor. Additionally, recruitment for the study was relatively easy—on the first morning of testing, we had more participants than we could accommodate. Anecdotally, several parents reported that they would not take children for venous sampling but appreciated the less invasive capillary blood draw. The procedure also appeared to be well tolerated by most of the tested children.

## 4. Discussion

This is a small pilot study based on a convenience sample of children living in Mount Isa. The geometric mean blood lead level among Indigenous children was nearly three-fold higher than that among non-Indigenous children. Additionally, the overall geometric mean in our sample (3.8 µg/dL (95% CI, 2.9; 5.0)) was higher than the geometric means reported by Queensland Medical Laboratory (3.0 µg/dL) and Mount Isa Hospital (3.2 µg/dL) in 2015, the most recent sampling for which results are available. Because our data are based on a non-representative convenience sample and a small number of observations, the geometric means reported here should be interpreted with caution. A larger, representative sample of Mount Isa children might find a higher or lower geometric mean blood lead level. It should also be noted, however, that Queensland Medical Laboratory and Mount Isa hospital data are also based on non-representative convenience samples and that the most recent Queensland Medical Laboratory data from 2015 is also based on a small number of tested children (*n* = 49). It is also not known how many Indigenous children have been tested in recent years, and a high proportion of Indigenous children in our sample with higher blood lead levels increases the overall geometric mean.

Despite the limitations of our sample, our findings should be considered in the context of limited information on blood lead levels in Mount Isa, particularly among Indigenous children. These findings are important from a public health perspective as they indicate that there are likely a significant number of children with blood lead levels ≥5 µg/dL in Mount Isa (40% in this sample), and most of these children have likely not been identified due to the low rate of blood lead level screening. Notably, of the 30 children who participated, 93% (28) of our sample reported never having been tested for lead despite many being, long-term residents in Mount Isa. All children with blood lead levels ≥5 µg/dL should be identified and provided with targeted public health intervention to reduce their exposure to lead. There are also likely to be additional children in Mount Isa with blood lead levels ≥10 µg/dL—our study identified three. These children urgently need to be identified so that public health intervention can occur. Priority should be placed on outreach, testing and public health intervention for Indigenous families as the majority of Indigenous children had blood lead levels ≥5 µg/dL.

The findings of this study support recommendations made by Forbes & Taylor [36] for a more proactive approach to reducing environmental lead exposure in Mount Isa. This should include the following elements: annual universal blood lead level testing of children followed by publicly reported results; culturally appropriate follow-up and intervention for children who are identified with blood lead levels ≥5 µg/dL; stack and fugitive emissions reductions; and other environmental strategies to reduce exposure to lead including remediation of soil contamination in children’s play and residential areas.

There is a compelling need to make annual universal screening of children standard practice in Mount Isa. Though increasing screening has been called for in Mount Isa for at least a decade, progress has been slow. The current approach to testing children (convenience sampling of a proportion of the population) is not a valid method for determining whether or not blood lead levels are increasing or decreasing in Mount Isa children. Ensuring that children’s health is being protected requires detailed and scientifically valid data on blood lead levels. Detailed reporting of results from universal screening would inform efforts to reduce environmental contamination and blood lead levels in the community.

Queensland Health leaders could look to the experience of Broken Hill where blood lead screening was incorporated with immunisation visits in 2011. Since then, there has been a marked increase in the proportion of children tested. Since 2012, approximately three out of four children in Broken Hill are tested on an annual basis, and detailed annual data reports guide public health efforts to reduce exposure [28].

The use of rapid point-of-care capillary testing, which is less invasive and provides immediate results, may reduce parental concerns about the discomfort to children from venous blood lead testing. Capillary testing can be used at clinic sites to screen all children annually at immunisation visits, which has been a successful approach in Broken Hill [40]. Screening can also be conducted annually at community daycare centres, and blood lead testing can be required for annual entrance to these locations. In September 2016, Queensland Health invested in four point-of-care test machines in Mount Isa (currently we understand these machines are in use at the Mount Isa Hospital, Gidgee Healing, and the Queensland Medical Laboratory). They credit the use of point-of-care screening for the increase in participation in blood lead testing in the past year. As the proportion of children screened increases, Queensland Health should be able to publish detailed results that can contribute to public health and environmental efforts to reduce children’s exposure.

Screening rates are likely to increase if parents do not feel stigmatised if their child has a blood lead level ≥5 µg/dL, and if parents perceive that there is real benefit to having their child tested—that is, if detection of blood lead levels ≥5 µg/dL is paired with tangible help. Stigma impeding testing has been an ongoing issue in mining and smelting communities, particularly when parents feel that they are to blame if their children have blood lead levels ≥5 µg/dL [41,42]. This may be attributable to health and industry officials’ over-reliance on health education messages related to personal hygiene, diet, and home cleaning, none of which have been shown to be efficacious in preventing lead exposure [43,44].

A concerted effort should be made to test all Indigenous children in Mount Isa as previous screenings have consistently found that Indigenous children are more likely to have blood lead levels ≥5 µg/dL, both in Mount Isa and in Broken Hill. In Broken Hill, the program partnered with Maari Ma, an Indigenous community health provider, and integrated testing into immunisation visits. These measures resulted in dramatically increased screening in the population with nearly all Indigenous children being tested annually.

Our prior research [27] found that public health messages aimed at reducing environmental lead exposure among children in Mount Isa do not have a strong focus on the Indigenous population and are not tailored to Indigenous families’ social and cultural backgrounds. Indigenous parents may have difficulty implementing all of the recommendations regarding cleaning, garden maintenance, and nutrition due to poverty and poor living conditions. Targeted and tangible assistance to Indigenous families, such as remediating contaminated residential soil and reducing bare earth in gardens, should be made available. Indigenous residents identified the lack of such tangible assistance as a barrier to blood lead level screening in Broken Hill [45].

The current focus on testing children ages 1–4 years old is limited and may provide a false sense of security to parents with younger and older children. While this is an at-risk age group, older children may also have blood lead levels ≥5 µg/dL. For example, in Herculaneum, Missouri, (US lead smelting city) in 2001, 8% of children ages 6–17 tested had blood lead levels >10 µg/dL [46]. Younger children aged 6–12 months should also be included in universal screening. Pregnant women, and women who intend to become pregnant, should also be tested.

Blood lead testing is secondary prevention—as it will not prevent lead exposure; however, it will provide health and environmental officials with valid data on the scope of the problem and can identify those at high risk so that assistance can be provided. Primary prevention approaches include reducing emissions and remediating existing contamination. These approaches must underpin efforts to reduce lead exposure.

Mount Isa’s lead emissions are considerable. Furthermore, allowable levels of lead in air exceed health-based standards set nearly a decade ago in the US. The current lead in air standard in Mount Isa is 0.5 µg/m^3^ averaged on an annual basis [47]. Annual averaging does not prevent short-term peak exposures, and an air lead level of 0.5 µg/m^3^ does not provide a margin of safety for protecting children’s health. For comparison purposes, after extensive scientific review, in 2008, the US EPA adopted a new lead in air standard of 0.15 µg/m^3^ (based on a three month rolling average) to protect children’s developing nervous systems and to prevent IQ loss, among other health effects [48]. In addition to an ambient standard for lead, in 2011 US EPA set an overall emissions limit for main stack emissions from primary lead smelters of 0.97 pounds or 0.4 kg of lead per ton of lead produced [49].

Pollution control devices at the Mount Isa smelting complex include a baghouse installed in 1992 at the lead smelter and an electrostatic precipitator for the copper smelter installed in 1994 [2]. Lead emissions in 2018 are projected to total as much as 83,544 kg or over 90 tons, with 93% coming from stack sources [50]. Upgrades to current pollution controls to reduce stack emissions are needed.

With respect to fugitive emissions, a 2015 emissions inventory shows—in order of decreasing contribution—that ore loading, unloading, and stacking, conveying transfer points, ore crushing, and wheel-generated dust are the activities resulting in the most fugitive lead emissions at the site [50]. All could be further controlled using the best available technology [51].

To address children’s exposure in homes and gardens, remediation of contaminated soil in residential areas, in children’s play areas and including school and daycare playgrounds is needed. In the US, removal and replacement of contaminated soil in residential gardens and areas regularly used by children is commonplace in communities near non-ferrous mining and smelting sites. For example, over 7000 properties have been ‘cleaned up’ in the Silver Valley of Idaho around a now disused lead–zinc mining and smelting complex. Clean-up has largely consisted of removing the top 6–12 inches of lead-contaminated soil and replacing it with uncontaminated soil [52]. Children’s blood lead levels have been substantially reduced due to environmental remediation efforts, with success attributed to cleaning individual gardens and neighbouring community properties [53,54]. In Broken Hill, a remedial approach with a focus on Indigenous children is being piloted. The 2015–2016 project plan dedicated $2.45 million toward a range of activities including remediation. The funding is part of a five-year $13 million NSW government commitment to addressing environmental lead exposure in Broken Hill [55].

## 5. Conclusions

This pilot study found that 40% of the children tested had blood lead levels ≥5 µg/dL. The proportion with blood lead levels ≥5 µg/dL was higher among Indigenous children as was the geometric mean blood lead level of these children. While our small sample size and reliance on convenience sampling do not permit extrapolation to the population of children in Mount Isa, it is clear that children with blood lead levels ≥5 µg/dL and ≥10 µg/dL are not being identified due to low rates of screening. Indigenous children appear to be at highest risk. Compared with other mining and smelting communities in Australia and in the US, there is an over-reliance on behavioural strategies (personal hygiene, diet, and in-home cleaning) that have not been found to be effective for preventing lead exposure [43,44]. A multi-component program of primary prevention (emissions reductions and environmental remediation) and secondary prevention (universal blood lead testing and culturally appropriate public health intervention) will help to address the chronic problem of lead exposure in Mount Isa.

## Figures and Tables

**Table 1:** Reported blood lead levels for children in Mount Isa, 1992–2017.

	Geometric Mean Blood Lead Levels, 95% CI (1–4 Year Olds); Children and Family Centre Testing (This Study) Includes 1–6 Year Olds			Percent 5.0 µg/dL or Greater	Percent 10.0 µg/dL or Greater	Minimum Maximum Blood Lead Level Values
	All Children	Non-Indigenous	Indigenous	All Children	All Children	All Children
Mount Isa Mines 1992–1994	10.9 (unspecified if geometric or arithmetic mean) (*n* = 101)	NA	NA	NA	36.7%	2–29
Queensland Health 2006/2007	5.0 (4.7, 5.2) (*n* = 400)	4.5 (4.3–4.8) (*n* = 315)	7.0 (6.2, 8.0) (*n* = 83)	NA	11.3%	1.3–31.5
Queensland Health 2010	4.27 (3.96, 4.61) (*n* = 167)	3.98 (3.68, 4.32) (*n* = 130)	5.44 (4.53, 6.53) (*n* = 37)	NA	4.8%	1.9–22.4
Queensland Medical Laboratory 2012	3.2 (*n* = 43)	NA	NA	21%	NA	NA
Queensland Medical Laboratory 2013	3.2 (*n* = 83)	NA	NA	23%	2.4%	NA
Queensland Medical Laboratory 2014	2.6 (*n* = 98)	NA	3.0	11%	0%	NA
Mount Isa Hospital 2014 (Aug.–Dec.)	3.2 (*n* = 57)	NA	3.5	16%	2%	NA
Queensland Medical Laboratory and other private labs 2015	3.0 (*n* = 49)	NA	3.9	14%	6%	NA
Mount Isa Hospital 2015	3.2 (*n* = 101)	NA	3.7	16%	2%	NA
Testing at Children and Family Centre March & May 2017	3.8 (2.9; 5.0) (*n* = 30)	2.4 (1.8, 3.1) (*n* = 16)	6.5 (4.7, 9.2) (*n* = 14)	40%	10%	1.6–17.3

Source: Data for Mount Island Mines was reported by Queensland Health [32]. All other data (excepting data from testing at the Children and Family Centre in March and May 2017) was reported by Queensland Health [22]. Queensland Medical Laboratory testing is reported as ‘Children under 5’. The lowest possible value for Queensland Medical Laboratory and Mount Isa Hospital is 2 µg/dL. The lowest value for Queensland Health 2006–2007 testing is 1.3 µg/dL. The lowest value for Queensland Health 2010 testing is 1.9 µg/dL. The lowest detectable value in the Children and Family Centre testing is <3.3 µg/dL; 1.6 µg/dL used in our analysis for all values <3.3 µg/dL. The number of Indigenous children tested is not available for Queensland Medical Laboratory and Mount Isa hospitals. Ninety-five percent confidence intervals reported where available. NA denotes data not available.

**Table 2:** Sample characteristics.

Age Distribution	Percent	*n*
1–2	20	6
2–3	30	9
3–4	26.7	8
4–5	16.7	5
5–6	3.3	1
6–7	3.3	1
Gender		
Male	40	12
Female	60	18
Indigenous Status		
Indigenous	46.7	14
Non-Indigenous	53.5	16
Type of Housing		
House	90	27
Apartment	10	3
Other	0	0
Child Previously Tested for Lead?		
Yes	6.7	2
No	93.3	28
Length of Time in Mount Isa (Months)		
Mean	33.1	30
Min–Max	10–53	30
Regular Play Outdoors		
Yes	90	27
No	10	3

**Table 3:** Blood lead level results.

Blood Lead Level Distribution	Percent	*n*
<3.3 µg/dL	36.7	11
3.3–4.9	23.3	7
5.0–9.9 µg/dL	30.0	9
10.0–14.9 µg/dL	6.7	2
15.0–19.9 µg/dL	3.3	1
Blood Lead Level by Indigenous Status	Geometric Mean (µg/dL)	
Overall sample	3.8 (2.9, 5.0)	30
Indigenous	6.5 (4.7, 9.2)	14
Non-Indigenous	2.4 (1.8, 3.1)	16
Blood Lead Level by Gender	Geometric Mean (µg/dL)	
Male	3.8	12
Female	3.8	18
Children with Blood Lead Levels ≥5 µg/dL	Percent	
Overall	40	12
Non-Indigenous	18.8	3
Indigenous	64.2	9
Male	41.6	5
Female	38.8	7
Ages 1–2	66.6	4
Ages 2–3	33.3	3
Ages 3–4	12.5	1
Ages 4–5	80	4
Ages 5–6	0	0
Ages 6–7	0	0

Ninety-five percent confidence intervals reported where available.
